# Knowledge and uptake of contraceptive and other sexual reproductive health services among in-school adolescents in three South African townships: Baseline findings from the Girls Achieve Power (GAP Year) Trial

**DOI:** 10.12688/gatesopenres.13636.3

**Published:** 2024-11-11

**Authors:** Melanie Pleaner, Alison Kutywayo, Mags Beksinska, Khuthala Mabetha, Nicolette Naidoo, Saiqa Mullick

**Affiliations:** 1 Wits RHI, Faculty of Health Sciences, University of the Witwatersrand, Johannesburg, Gauteng, 2193, South Africa; 2 MRU (MatCH Research Unit), Department of Obstetrics and Gynaecology, University of the Witwatersrand, Durban, Kwa zulu Natal, 4001, South Africa

**Keywords:** Adolescents, Contraception Use, South Africa, SRH Knowledge, Uptake

## Abstract

**Background:**

South African adolescents experience barriers to sexual and reproductive health (SRH) knowledge and uptake. This study provides insight into contraceptive and other SRH service knowledge, perceptions, and uptake among adolescents in high HIV prevalence settings.

**Methods:**

A baseline cross sectional survey was conducted among 3432 grade 8s enrolled into the Girls Achieve Power (GAP Year) trial from 26 public high schools across three South African townships (Soweto, Thembisa and Khayelitsha) (2017 - 2018). An interviewer-led survey collected information on SRH knowledge and perceptions; an audio computer-assisted self-interviewing technique gathered SRH service uptake. Descriptive analysis indicates frequency distribution of socio-demographics and knowledge, uptake and perceptions of SRH services. Chi-square test tested for associations between age and sex and selected variables that measure SRH knowledge and uptake.

**Results:**

In total, 2383 participants completed both survey components. Of these, 63.1% (n=1504) were female and 81.4% (n=1938) aged 12-14. Almost a fifth (18.3%, n=436) had ever had sex and less than 1% had accessed SRH services in the last year. Of the 157 females who had ever had sex, 50.9% had ever used contraception. Of those who had sex in the last three months, 59.0% reported using a contraceptive method. Condom use was inconsistent: almost all females said they had not used or could not remember if a condom was used at last sex.

**Conclusion:**

This paper contributes to the evidence strengthening learner SRH education, including the national Integrated School Health Programme. Key themes include the need for age-appropriate, differentiated comprehensive sexuality education (CSE) for the range of ages found in the same grade in South African schools. Education on different contraceptive methods, informed decision-making, and emergency contraception is key. School-based interventions should embrace integrated HIV, STI, and pregnancy prevention messages. Closer links with health services need to be constantly fostered and reinforced.

## Introduction

Globally, there has been significant focus on the potential dividends to be gained by investing in and prioritising adolescent health, including sexual and reproductive health (SRH) and HIV. Although great strides have been made since the SRH historical landmark - the International Conference on Population and Development (ICPD) in 1994 - there are still multiple challenges in accessible SRH service provision for adolescents, resulting in poor health outcomes ^1, 2^. This is particularly so for adolescent girls and young women (AGYW) in sub-Saharan Africa, who continue to experience significant inequalities, challenges and unmet needs for SRH services, together with an associated increased risk of unintended pregnancies, HIV, STIs, complications related to early age pregnancy, and other SRH complications ^3, 4^. In sub-Saharan Africa, more than 50% of rural AGYW aged 15–24 years, and 42% of those in urban areas have been pregnant before the age of 18, with one in five new HIV infections occurring in this age group, despite representing only 10% of the population ^5^. This is primarily attributed to limited information, barriers to access, constraints in exercising their SRH rights, lack of comprehensive programmes and service provision, and in many countries, framed by an unsupportive legislative and policy environment ^6, 7^.

Although South Africa has progressive and enabling rights-based laws, policies, guidelines and programmes relating to SRH and young people ^8– 10^, the challenges faced in relation to SRH and HIV mirrors similar trends in sub-Saharan Africa. In terms of HIV, 7% of young people age 15–24 are HIV-positive, with AGYW having a four times higher prevalence rate than their male counterparts (12% versus 3%) ^11^. The most recent South African National HIV Prevalence, Incidence, Behaviour and Communication Survey ((SABSSM V) ^12^ provides insight into some of the most important drivers of HIV in young people in South Africa, including early sexual debut, age disparate sex, and condom use. In terms of sexual debut in young people who have had sex, 13.6% reported having had sex before the age of 15 years. More males (aged 15–24) reported this compared to females (13.6% males vs 7.6% females). This pattern has been consistent over the previous four surveys ^12^.

Teenage pregnancy and its consequences are an ongoing problem ^13, 14^. The 2016 South African Demographic and Health Survey (SADHS) found that 15.6% of South African adolescent females aged 15–19 have begun childbearing. Although adolescent pregnancy has been a major social and economic challenge in South Africa for decades, the prevalence of unintended pregnancies among females aged 15–19 has remained unchanged for the last 20 years ^11^. The ramifications of unsupported teenage pregnancy are far reaching and have been well documented, and include early exiting from education ^15^, reduced opportunities in employment and further education, socio-economic challenges, associated health risks for both the woman and the infant, increased vulnerably for STIs, HIV and gender-based violence, together with the need for social protection and support ^14– 17^.

Contraceptive prevalence in sexually active adolescents aged 15–19 was 60.4% in the 2016 SADHS, with reliance primarily on injectables and male condoms ^11^. However consistency in condom use in this age group is poor ^12^ leaving young women open to unintended pregnancy. Factors contributing to poor contraceptive uptake in adolescents are cross cutting and multi-fold, and include health inequalities, limited contraception knowledge, barriers to accessing services, challenges with regards continuation and correct use, and gender-based violence ^18– 20^.

Knowledge about contraception and various contraceptive options is low among South African youth, particularly in relation to both long-acting reversible contraceptives and emergency contraception. For example, data from the 2012 South African household survey indicated only 30.9% of 15–19-year-old females knew of intrauterine devices (IUD), and 36.2% of emergency contraception ^21^. A school-based study focusing on secondary school girl learners in grades 10–12 ^22^ showed that while there was awareness about methods, this information was superficial - 58% knew about condoms, 50% injections, 43% oral contraceptive pills, 40% female condom and 10% IUDs, and only 17% knew about emergency contraception, but did not know how to take them. Similarly, there was a lack of information about how to take oral contraceptive pills ^22^. In this manuscript knowledge refers to awareness of both services and of methods used.

The need to use evidence to understand and design effective, responsive strategies to improve the provision of SRH services for AGYW, and more specifically, preventing teenage pregnancy and the promotion of contraception is now as urgent as ever. However, there is still a gap in research relating to adolescents' use and knowledge of contraception and related services ^23^. This study seeks to contribute to the evidence and understanding of the factors influencing adolescent contraception uptake. To this end, we aim to gain insight into the knowledge and uptake of contraception among grade 8 adolescents in a high HIV prevalence setting and utilise the findings to guide interventions to improve access to SRH services, inform comprehensive sexual education (CSE) and strengthen strategies to prevent unintended pregnancies, within the framework of the Integrated School Health Programme (ISHP).

## Methods

### Study design and setting

This research is part of the Girls Achieve Power Trial ( GAP Year) cluster randomized controlled trial (cRCT) conducted in 26 non-fee-paying high schools, in three peri-urban townships of South Africa: Khayelitsha (Western Cape Province), and Soweto and Thembisa (Gauteng Province) ^24– 28^. GAP Year was seeking to test the effectiveness of a CSE asset-building intervention aiming to reduce school dropout among adolescent girls between grades 8–10 while shifting gender attitudes and encouraging positive behaviour change among adolescent boys ^27^. The GAP Year intervention adopted a four-pronged approach, across the ecological model: a sports-based after-school intervention, parent intervention which includes dialogues and text messaging, linkage to care and school safety ^27^. Half of the schools were randomised to the study arm, whilst the other 13 were in the control arm. Schools were selected using the following inclusion criteria: mixed sex public high schools in in quintiles 1–3 ^*^ which had not been exposed to any asset building interventions in the past six months. The exclusion criteria for the study were: single-sex schools; private schools; schools that have been exposed to similar interventions and public schools that cater for learners with special needs. A baseline cross sectional analysis was conducted for grade 8 learners enrolled in GAP Year to assess the knowledge, patterns and uptake of contraception among adolescent learners. Data collection took place between April 2017 and September 2018 for all 26 schools.

### Study population

All grade 8 adolescents at selected schools, irrespective of age, sex or race, were invited to participate. The grade 8 learner age range is approximately 12–14 years however due to learners repeating grades and other reasons, the age range is commonly wider with older learners enrolled up to 18 years ^29, 30^.

### Sample size justification

The sample size was computed using cluster-randomized size methodology, suggested by Hayes and Bennett ^31^, based on the study’s primary outcome measures, (school dropout and increased reporting of GBV among adolescent girls). The effect size of dropout was factored from other similar studies accounting for a large conservative and representative sample size to measure outcomes. Based on other local studies, it was hypothesized a reduction in drop-out rate from 17.8% as reported by Branson, Hofmeyr ^30^ to less than 14% (estimated effect size of 20%), with an anticipated attrition rate of 5% per year based on a similar local study ^32^. These calculations resulted in an upper limit sample size of 2730 adolescent girls and 1850 boys to determine the association between intervention and control school’s dropout rate and GBV variables, which reflects the sample of the actual trial.

### Measures

The baseline survey was comprised of two components: firstly, an interviewer-led questionnaire and secondly, an audio computer-assisted self-administered interview (ACASI). The first section lasted between 45 minutes to 1 hour, providing information on the participants’ demographic and socio-economic characteristics, knowledge and attitudes pertaining to school safety, social support and social networks, sexuality, gender and norms, sexual reproductive health and rights (SRHR) and care-seeking behaviours. It was conducted by trained fieldworkers and captured directly on an android tablet, formatted with the Research Electronic Data Capture (REDCap) system ^33^. In the second section, administered using the Audio Computer Assisted Self-interviewing (ACASI) method, participants were asked sensitive questions regarding their actual practices and behaviour, including questions on uptake of health care services, contraceptive use, sexual debut and relationships. This section lasted 20–30 minutes. The ACASI method was adopted, was seeking to reduce social desirability bias, and later exported into the REDCap online system. The survey was developed in English and back translated into Xhosa (a commonly spoken language at one site) and then pre-tested among selected participants for comprehension. Participants indicated that they preferred to complete the survey in English and therefore translation for the other sites was not provided. During data collection, participants were assigned unique person identifiers. As such, names and other personal information of the participants were not revealed in the datasets.

The data were analysed at two levels. Descriptive analysis was used to show the frequency distribution of the sociodemographic characteristics of the respondents as well as the knowledge, uptake and perceptions of sexual and reproductive health services. At the bivariate level, a chi-square test of association was employed to test for an association between age and sex categories and selected variables that measure sexual and reproductive health.

### Data management and analysis

The tablets that were used to collect data utilised password-protected mechanisms to protect the data and the synced data was stored on Wits RHI secured servers. All the data from the REDCap and ACASI systems was exported into Stata 17 ^34^ for analysis. At the univariate level, descriptive frequency tables were used to describe the socio-demographic characteristics of the participants by age and sex. At the bivariate level, variables measuring knowledge, uptake and perceptions around SRH were assessed to examine their association with both sex and age category. Participants’ age was presented in groups (12–14 years and 15–18 years), this is because the 15–18-year age group represent a group that are older than the standard age appropriate range for that grade. They comprised of those who have repeated or skipped a year of schooling or started school later. They may represent a more vulnerable group whose education has been affected by social or economic circumstances. It should be noted that the “no” category has been removed for all dichotomous variables [variables with a Yes or No Response] shown in the results tables.

### Ethical approval and considerations

The study was approved by the University of the Witwatersrand Human Research Ethics Committee (#M160940) in September 2016. The study was also approved by the provincial research committees of the Western Cape and Gauteng Departments of Health and of Education. This was followed by the schools’ approval and written parental informed consent and learner assent. The participating schools, parents and participants were fully informed about the voluntary nature of participation in the study, and of the confidentiality of data management. All data collection was supervised by the research team. Interviewer environments were set up to ensure confidentiality. Where feasible, interviewers were the same sex as the learner. Participants could stop the interview process at any time and were free to refuse to respond to any question(s) they felt uncomfortable answering. Social workers were employed to provide psychosocial support to participants during data collection and study intervention and a social harm form was developed to facilitate prompt referrals, where needed. The Good Participatory Practice Framework was adapted and adopted in the GAP Year trial to guide stakeholder engagement throughout the lifecycle ^27^.

## Results

As previously reported ^24– 26, 28^, overall, 3432 eligible participants across 26 schools participated in the baseline survey: we included 2383 in the analysis who completed both sections of the survey. In some cases, due to lack of time, participants were unable to complete both components and were excluded from this analysis.

Table 1 provides the socio-demographic characteristics of participants, by sex and age group. As previously reported ^24– 26, 28^, of the 2383 participants, 63.1% (n=1504) were female and the majority were Black African (96.9%, n=2309). Overall, Gauteng province represented just over half of all participants (53.6%, n=1278), with more males than females (57.0% vs 51.7%, p=0.012). Almost one in five participants (18.1%, n=433) in grade 8 were older than 14 years with 5.1% 16 years and older. Under half of all participants (41.4%, n=967) reported living with both parents. Over two thirds of participants’ parents/guardians’ households were receiving government grants (67.3%, n=1498).

**Table 1.** Socio-demographic characteristics of participants, by sex and age category.

	Sex			Age groups ^1^			Total
	Female (n=1504) % (n)	Male (n = 879) (n)	P-value	12–14 (n = 1938) % (n)	15–18 (n =443) % (n)	P-value	(n=2383) % (n)
**Racial group**							
African	96.8 (1456)	97.0 (853)	0.751	96.7 (1874)	97.7 (433)	0.253	96.9 (2309)
Coloured	3.2 (48)	3.0 (26)		3.3 (64)	2.3 (10)		3.1 (74)
**Province**							
Western Cape	48.3 (727)	43.0 (378)	**0.012**	44.5 (863)	54.6 (242)	**<0.001**	46.5 (1105)
Gauteng	51.7 (777)	57.0 (501)		55.5 (1075)	45.4 (201)		53.6 (1278
**Lives with**							
Both parents	39.4 (580)	44.8 (387)	0.018	42.2 (803)	38.0 (164)	0.006	41.4 (967)
Single parent	41.5 (611)	39.7 (343)		41.3 (785)	39.1 (169)		40.9 (954)
Relative/guardian	19.0 (280)	15.5 (134)		16.5 (314)	22.9 (99)		17.7 (414)
*Not stated*	*33*	*15*		*36*	*11*		*48*
**Parent/guardian employed ^2, 3^**	66.9 (1002)	72.0 (631)	** 0.010**	69.6 (1344)	65.2 (288)	0.069	68.8 (1633)
**Parent/guardian receives ** **government grant**	68.7 (965)	64.9 (533)	0.068	66.3 (1202)	71.4 (294)	0.050	67.3 (1498)
* Don’t know*	*99*	*58*		*126*	*31*		*157*
**Dating or in a relationship**	46.7 (703)	59.3 (521)	**0.000**	48.4 (938)	64.1 (284)	**0.000**	51.4 (1224)
**Ever had sex ^4^**	10.4 (157)	31.7 (279)	**<0.001**	15.0 (291)	32.7 (145)	**<0.001**	18.3 (436)
**Age of sexual debut ^5^ (yrs)**							
7–10	7.0 (11)	21.8 (56)	**<0.001**	19.0 (52)	10.7 (15)	**<0.001**	15.4 (67)
11–14	69.2 (108)	64.6 (166)		75.8 (207)	47.9 (67)		62.8 (274)
15–17	23.7 (37)	13.6 (35)		0.0 (0)	37.3 (72)		6.2 (72)

^1^ *Missing age category n=2* ^2^ *At least one parent or guardian employed* ^3^ *Parent or guardian employed missing n=8* ^4^ *Ever had sex missing n=3* ^5^ *Age of sexual debut not disclosed n=23*

Just over half of participants (51.4% n=1224) reported that they were dating or in a relationship. Almost a fifth (18.3%, n=436) had ever had sex, with significant group differences by age group and sex (p=0.001). While fifteen percent of those aged 12–14 years reported ever having sex, this more than doubled (32.7%) in the 15–18 year olds. Almost two-thirds (62.8%) were aged 11–14 years when they first had sex.

## Knowledge, uptake and perceptions of sexual and reproductive health services

Table 2 outlines knowledge, uptake and perceptions of SRH services, by sex and age group. Although over two thirds of participants (66.8%, n=1557) perceived that they have a right to access health care services without being discriminated or stigmatized by health workers, this was significantly different between male and female participants (p=<0.001).

**Table 2.** Knowledge, uptake and perceptions of sexual and reproductive health services, by sex and age category.

	Sex			Age groups			Total
	Female (n=1504) % (n)	Male (n = 879) % (n)	P-value	12–14 (n = 1938) % (n)	15–18 (n =443) % (n)	P-value	(n=2383) % (n)
**Have a right to access healthcare without being discriminated or stigmatized ** **by a health worker (n=2332)**	65.2 (965)	69.5 (592)	**<0.001**	66.8 (1269)	66.4 (287)	0.504	66.8 (1557)
**Ever participated in an SRH programme (n=781)**	37.8 (557)	26.3 (224)	**<0.001**	32.5 (615)	38.3 (165)	**0.006**	33.6 (781)
**Place where you participated in this programme (n=777)**			**<0.001**			**0.285**	
School	75.0 (415)	71.0 (159)		74.5 (455)	71.5 (118)		73.8 (573)
Church	12.5 (69)	25.0 (56)		15.2 (93)	19.4 (32)		16.1 (125)
Private organisation	9.6 (53)	0.4 (1)		7.5 (46)	4.8 (8)		7.0 (54)
Other	2.9 (16)	3.6 (8)		2.8 (17)	4.2 (7)		3.1 (24)
**Participated in a SRH programme in last ** **2 years (n=781)**	36.8 (205)	36.6 (82)	0.959	35.8 (220)	40.0 (66)	0.317	36.7 (287)
**Know of a place in community where ** **young people can find out about SRHR ** **(n=2335)**	30.9 (455)	32.2 (277)	0.184	29.6 (561)	39.2 (171)	**<0.001**	31.4 (732)
**Accessed health care in the last year ** **(n=2337)**	56.6 (836)	59.5 (512)	0.166	58.4 (1108)	54.8 (239)	0.178	57.7 (1348)
SRH Health services required ^a^							
Sexually transmitted infections (STIs)	0.7 (17)	0.5 (12)		0.5 (20)	1.0 (9)		0.7 (29)
HIV testing services (HTS)	0.5 (11)	0.5 (13)		0.4 (17)	0.8 (7)		0.4 (24)
Pregnancy test	1.2 (29)	N/A		1.0 (19)	2.3 (10)		1.6 (29)
Contraceptives	2.6 (61)	0.4 (10)		1.1 (44)	3.0 (27)		2.1 (71)
Injury	3.9 (88)	4.6 (105)		4.1 (158)	3.9 (35)		4.0 (193)
Non-SRH Services	36.5 (789)	23.2 (461)		27.4 (1061)	21.2 (189)		24.3 (1250)
**Health care site accessed at last visit (n=1345)**							
Youth clinic	3.6 (30)	1.8 (9)	**0.025**	2.6 (29)	4.2 (10)	0.464	2.9 (39)
Private clinic/ hospital	6.0 (50)	9.0 (46)		7.2 (79)	7.1 (17)		7.1 (96)
Government/ public clinic	89.6 (748)	87.7 (447)		89.2 (986)	87.0 (208)		88.9 (1195)
Other	0.8 (7)	1.7 (8)		1.0 (11)	1.7 (4)		1.1 (15)
**Felt comfortable to ask questions at my last visit (n=1324)**	51.7 (426)	46.6 (233)	0.072	51.4 (559)	42.1 (99)	**0.010**	49.8 (659)
**Questions asked at last consultation were answered adequately (n=1304)**	48.2 (391)	47.5 (234)	0.793	49.5 (529)	40.4 (95)	0.011	47.9 (625)
**Enough confidentiality at last visit (1315)**	34.7 (283)	31.9 (159)	0.294	33.1 (356)	35.9 (85)	0.407	33.6 (442)
**Requested contraceptive services at last visit (n=1757)**	26.2 (16)	N/A		27.8 (10)	24.0 (6)	0.741	26.2 (16)
**Main source of SRHR information ^b^**							
School teacher	58.1 (874)			58.3 (746)	56.7 (127)		58.1 (873)
Mother	38.9 (585)			40.0 (512)	32.6 (73)		38.9 (585)
Television	12.2 (183)			12.7 (162)	9.4 (21)		12.2 (183)
Friends	10.6 (150)			10.2 (131)	13.0 (29)		10.6 (160)
Sister	10.4 (157)			9.9 (127)	13.4 (30)		10.4 (157)
Other family members	9.6 (144)			9.5 (121)	10.3 (23)		9.6 (144)
Books/ magazines	7.2 (108)			7.7 (99)	4.0 (9)		7.2 (108)
Healthcare workers	6.5 (97)			6.4 (82)	6.7 (15)		6.5 (97)
Radio	4.6 (70)			4.8 (61)	4.0 (9)		4.6 (70)
Father	3.5 (53)			3.4 (44)	4.0 (9)		3.5 (53)
Online	3.5 (53)			3.8 (49)	1.8 (4)		3.5 (53)
Films / videos	2.1 (31)			2.4 (31)	0.0 (0)		2.1 (31)
Brother	1.7 (25)			1.6 (21)	1.8 (4)		1.7 (25)
Other	2.5 (44)			3.0 (38)	2.7 (6)		2.5 (44)

*^a^Multiple response answer* *^b^Multiple response answers, question not asked of males*

A third (33.6%, n=781) had ever participated in an SRH programme in the past 2 years ^†^, with males (p<0.001) and those aged 12–14 years (p=0.006) more likely to have participated than females and those 15 –18 years. Almost three-quarters participated in the SRH programme in their school. Far fewer had participated in an SRH programme in the last two years (12.0%, n=287).

Less than a third of all participants knew of a place in their community where they could access SRH information, with older adolescents (15–18 years) more likely to know where to access this information (39.2% vs 29.6%, p=0.001).

Over half of participants (57.7%, n=1348) had accessed healthcare in the past year, with most requiring non-SRH services. Less than 1.0% accessed HIV or STI services, and a small number (2.1%, n=71) accessed contraception. Most participants used public health clinics (88.9%, n=1195), with only 2.9% (n=39) having accessed a youth clinic at their last visit. During their last healthcare visit, almost half (49.8%, n=659) felt comfortable enough to ask questions, with 47.9% (n=625) having their questions answered adequately. In relation to rights, only one third (33.6%, n=442) felt there was enough confidentiality during their last visit. The main source of SRH and rights education in females were school teachers (58.1%, n= 873) and mothers (38.9%, n= 585).

## Uptake and patterns of contraceptive use in females

Table 3 reports on contraceptive history and current use in females who had ever had sex. Males are not presented as most were unsure about their partners current contraception method use. Of the 157 females who had ever had sex, half (51.0%, n=80) had ever used a contraceptive method. Due to the small sample of females who had ever used a contraceptive, Table 3 presents descriptive statistics by age group. The injection and condoms were the most common method ever used in the 12–14 age group. Almost all (96.4%, n=27) of those aged 15–18 years had ever used the injection. Of the whole sample, 36 females were current contraceptive users, although 29 of these had not had sex in the last three months.

**Table 3.** Uptake and patterns of contraceptive use of females who have ever had sex, by age.

		Age groups	Total
	12–14 (n=109) % (n)	15–18 (n = 48) % (n)	(n=157) % (n)
**Ever used contraceptives**	46.8 (51)	60.4 (29)	51.0 (80)
**Contraceptive method ever used ^a^**			
Injection	63.3 (31)	96.4 (27)	75.3 (58)
Condoms	59.2 (29)	21.4 (6)	45.4 (35)
Pill	4.1 (2)	7.1 (2)	5.2 (4)
Emergency contraceptives	2.0 (1)	3.6 (1)	2.6 (2)
Implant	4.1 (2)	0.0 (0)	2.6 (2)
Vaginal ring	2.0 (1)	0.0 (0)	1.3 (1)
Intrauterine Device (IUD)	2.0 (1)	0.0 (0)	1.3 (1)
Traditional methods ^b^	3.92 (2)	0.0 (0)	2.6 (2)
**Sexually active in last three months (N=61)**	38.8 (40)	46.7 (21)	41.2 (61)
**Current contraceptive method use (N=36)**	57.5 (23)	61.9 (13)	59.0 (36)
Condoms	69.6 (16)	38.5 (5)	58.3 (21)
Injection	56.5 (13)	92.3 (12)	69.4 (25)
Implant	4.3 (1)	0.0 (0)	2.8 (1)
Intrauterine device (IUD)	0.0 (0)	7.7 (1)	2.8 (1)
Emergency contraception	0.0 (0)	7.7 (1)	2.8 (1)
Dual method ^c^	40.8 (7)	25.8 (5)	33.3 (12)
**Condom use at last sex** No	0.0 (0)	0.0 (0)	0.0 (0)
Can’t remember	22.5 (9)	14.3 (3)	19.7 (12)
**Know that condoms** **can prevent HIV and STIs**	84.4 (38)	92.6 (25)	87.5 (63)
**It was my choice to start using contraception (n=80) ^d^**	2.5 (1)	4.5 (1)	3.2 (2)
**If no, who made the decision for you (n=60)**			
Parents	100.0 (39)	100.0 (21)	100.0 (60)
**Ever discussed contraception with my partner (n=157)**	0.0 (0)	0.0 (0)	0.0 (0)
**Method used (Self/partner) to prevent pregnancy and STIs at first sex (n=157)**	56.3 (58)	55.6 (25)	56.1 (83)
**Knowledge of where to get contraceptives (n=157)**	82.4 (89)	83.3 (40)	82.7 (129)
**Can obtain contraception without my parents’ permission (n=157)**	27.4 (29)	22.9 (11)	26.0 (40)

^a^ Of those who are currently using contraception, they had a multiple response option to select their current forms of contraception ^b^ Traditional methods refer to withdrawal and thigh sex ^c^ Condoms only or hormonal method and condoms ^d^ 18 missing responses

Of the 157 females who had ever had sex, 41.2% (n=61) reported having sex in the last three months. Of these 59% (n=36) reported current contraceptive use with two-thirds (69.4%, n=25) using the injection with close to 60% using the condom (58.3%, n=21). Almost half of the injectable users mentioned using condoms as an additional method but not one of these or the condom only group reported use of a condom at last sex. Most knew condoms could prevent HIV and STIs. A small proportion mentioned hormonal methods (implants, injections and oral pills) could prevent HIV and STIs. Similarly, a small number (2.8%) mentioned the vaginal ring.

Of the 80 who had ever used contraceptives, only two said it was their decision to start using a method. Sixty said it was their parents/guardians who made the decision for them, while 18 did not say who had made the decision. None of the females who had ever had sex had ever discussed contraception with their partner.

At the first sexual encounter, just over half (56.1%, n=83) did something to prevent pregnancy, HIV or sexually transmitted infections. Most (82.7%, n=129) knew where to get contraceptives but fewer (26.0%, n= 40) knew that they could get contraceptives without their parents’ permission.

Of the 80 females who have ever had sex and ever used a contraceptive method, the majority got the method from a public/government clinic (33.8%, n=26), followed by a private clinic (23.4%, n=18), pharmacy (10.4%, n=8), corner shop (6.5%, n=5), and friend (5.2%, n=4), while some did not say where they had obtained their method. Of those who reported they had never had sex, a small proportion reported using a contraceptive method.

Five females, of the 157 who had ever had sex, had ever been pregnant (0.3%, n=5): two got pregnant at 14 years, one at 15 years and two at 16 years (p=0.001). Of those who had ever been pregnant, two were pregnant at the time of the survey, one had terminated the pregnancy and two had gone on to have a live birth.

## Discussion

We set out to explore adolescent experience with SRH service knowledge and uptake, in particular contraception, in three peri-urban settings in South Africa. Although the data collection was undertaken in one school grade, a considerable proportion were above the standard expected age for the grade, this has been previously reported ^35^. These older participants are known to experience a number of challenges ^30, 35, 36^, and may be missing out on age-appropriate school-based CSE which would be received if they were in a higher grade.

In terms of sexual experience, over half of the participants were dating or in a relationship and just under a fifth, (18.3%) had ever had sex. Two-thirds had their first sexual experience between the ages 11–14 years, and a small number below this age. Of those who had had sex, many did nothing or did not remember if a method was used to prevent pregnancy, HIV or STIs during that first sexual encounter. This highlights the importance of programmes focussing on early adolescents, including age appropriate SRH information to be provided ensuring adolescents, including very young adolescents, are equipped with the knowledge around safe sexual practices, rights and gender awareness ^37, 38^. The GAP Year afterschool intervention sought to address this gap in knowledge ^27^. Interestingly, participants reported that parents were commonly involved in their decision to use contraception – this is a potential area for further research.

Although over half the participants reported having accessed healthcare services in the last year, only around 1% or less in the 12–14 age group reported they required SRH services, and this was only slightly higher (up to 3%) in the older age group. Additionally, less than 5% attended a dedicated youth service for a service. The low reporting of SRH services may be simply a reflection of the demand for services in a population where under a fifth had ever had sex, and of these only a small proportion had ever used contraception, had an HIV or a pregnancy test. The latest SADHS ^11^ indicated that the most popular method used by adolescents is the male condom and condoms are available outside of public health sector services. Of those who had used healthcare services, they commonly perceived a lack of confidentiality and the ability to ask questions, and were concerned about discrimination, this was especially so for those who mentioned healthcare workers as their primary source of SRHR information. The older age group reported being less comfortable about asking questions- at their last healthcare visit. However this may be due to the higher proportion of sexually active participants in this age group who may have wanted to ask more questions on SRH issues. Addressing barriers to health care is an integral part of SRH promotion ^39^, highlighting the need to improve young people’s access to youth friendly services from a rights-based perspective.

Given the low knowledge and uptake of youth-friendly SRH services in this study, dismantling barriers, improving access, and developing effective responses to adolescent SRH and contraceptive programming is vital. Studies show that high impact interventions include improved access to youth friendly services ^40^, school-based interventions ^41^, and in particular well designed, and age-appropriate CSE ^42, 43^, focusing on early adolescents, where patterns of behaviour are being formed ^44^. The provision of quality, youth-friendly contraceptive services, sensitive to and responsive to the needs of young people is a fundamental requirement for promoting the SRH of young people ^45^, as well as services that promote informed choice and decision-making, including long acting reversible contraception in the method mix ^46^. In addition, the need for interventions targeting adolescent males as well has been underscored ^47^.

School teachers and mothers were noted as the most common source of SRH information. While the survey did not ask about the satisfaction of the participants with information from these specific sources, it highlights two important priorities – to equip parents with quality knowledge and skills to communicate effectively with their children about sex, and the need to provide teachers with the knowledge and skills to provide information, supported by comprehensive curricula and policies. Interventions focussing on parents have been shown to be constructive but neglected and should be included in programmatic interventions ^48, 49^. This finding reinforced the importance of including parent dialogues and events in the GAP Year intervention ^27^. In South Africa, in 2010 the Department of Education introduced scripted lesson plans to strengthen the SRH content and implementation of the Life Orientation Curriculum ^50^. Despite the support school teachers received, no impact was observed on the primary outcomes of HIV knowledge, attitudes, condom use and pregnancy incidence ^50^. These findings question whether this was due to the programme being ineffectively designed or challenges with programme implementation and lack of programme fidelity ^51, 52^. The National Integrated School Health Policy ^8^ outlines what areas should be included in the school health package per learner phase. Contraception is included in the two senior phases from grade 9–12, however there are no further details or guidance given. The Standard Operating Procedures for the Provision of Sexual and Reproductive Health, Rights and Social Services in Secondary Schools ^9^ mention the need to counsel on the full range of available methods as laid out in the National Contraception Policy ^53^.

There was similar contraceptive ever and current use in sexually active females to previously reported data in this age group ^11^. It was interesting to note that a number of females who had not had sex in the last three months reported that they were using a contraceptive method as were some who reported never having had sex. Although this was not probed in more detail, this may reflect an intention to have sex, or prevention in the event that they have sex. This, too, is an interesting trend for further research.

Contraceptive method use was similar to national surveys in South Africa with injectables and condoms as the main methods of contraception ever and currently used in sexually active participants ^11^. These method choices reflect those of all women in South Africa where injectables are the most popular method of contraception ^11^. The hormonal implant was introduced in 2014 and uptake was low in the first few years, however, it is now the second most popular method to the injectable DMPA ^54^. Whilst we didn’t directly assess knowledge on various contraceptive methods, it highlights the need to promote all available contraceptive options, particularly the use of long acting, reversible contraceptive methods which are both highly effective, do not rely on regular visits to the clinic, nor clients remembering to take them daily ^55^. In addition, only two girls mentioned having ever used emergency contraception, and noting that only half who had had sex had ever used contraception, the need to promote emergency contraception is also an important, but underused option ^56^. The Standard Operating Procedures for the Provision of Sexual and Reproductive Health, Rights and Social Services in Secondary Schools ^9^ clearly states the need to inform learners of emergency contraception and where it is available. These guidelines should be specifically targeted to educators involved in school SRH programme delivery.

There are several factors associated with contraceptive uptake, and these all need to be accompanied by strengthened adolescent and youth friendly services and improved outreach concerning available SRH and HIV prevention and services – for example, it was shown that some participants thought the hormonal contraception could prevent HIV and STIs, the majority of participants reported not using protection at sexual debut, and a very low percentage used condoms at last sex. This calls for ongoing messaging concerning dual protection and the promotion of condom use and other HIV and STI prevention options, such as oral PrEP.

This study endorses existing literature which shows awareness of contraception and contraceptive services is low ^21, 22^. This is important because knowledge, access to, and use of contraception plays a significant role in averting and decreasing millions of unintended pregnancies, births, abortions, and maternal deaths each year. Improving knowledge about contraception, as well as an understanding of their rights can help AGYW make informed decisions that can positively impact their SRH, education and psychosocial well-being ^40, 57^.

This all serves to highlight the need for school-based interventions that work across the ecological model ^27^ - empowering individuals; promoting supportive relationships with sexual partners ^58^, parents, and peers and teachers and at the community level.

## Strengths and limitations

There are strengths and limitations that should be considered when reviewing these findings. Whilst this study was conducted in 26 schools in three highly populated diverse townships of South Africa, its generalizability is limited to similar contexts. The study was cross sectional therefore only representing one point in time. The final study sample size was less than originally planned however the prevalence of contraceptive use and method mix reflect that of the most recent South African demographic health survey ^11^. Participation was voluntary, however the refusal rate was not collected which may have potentially introduced a participation bias. There were many participants who didn’t complete both components of the survey which reduced the sample size when analysing specific variables. There was a higher proportion of participants aged 12–14 years in relation to those aged 15–18 years increasing power which results in these significant age differences: therefore, the results should be interpreted with caution given that the proportion of participants in these age groups is unbalanced. There were some significant differences presented in the results in relation to ‘ever had sex’ and ‘ever participated in an SRH program’ which could be attributed to underreporting of sexual activity and the targeting of AGYW in SRH programs.

## Conclusion

This paper aimed to build on the body of evidence to guide school-based interventions to improve the SRH of school-going participants. The paper highlights the need to encourage health seeking behaviour and promote the idea that health services not only for problems but important for prevention and health promotion - especially in relation to sexual health, including HIV, STI and pregnancy prevention. This means the importance of knowing where services are located, as well as building partnerships with public health clinics to ensure that services are accessible and responsive to the needs of young people. The importance of school teachers and parents as the primary source of SRH information for learners requires an expanded, enhanced, programme to equip both teachers and parents to provide accurate, relevant and accessible information. Several points related to life orientation, life skills and CSE were underscored including the need for age-appropriate, differentiated approaches to cater for the range of ages, and particularly for over-aged learners, in South African schools; the need for CSE to start with early grades; as well as the need to deal with issues rooted in young people’s realities and sexual lives – such as communicating with sexual partners about safer sex, using protection at first sexual encounters (and thereafter) and the ongoing need to negotiate and use condoms. In addition, the need for education in relation to contraceptive options and sexual and reproductive health rights was highlighted.

## Consent

Participants and their parents or guardians provided written informed consent for the publication of this data.

## Data Availability

These data are comprised of aggregated survey responses, data codebooks and the survey tool. These data are available from: This project contains the following underlying data, found at: ", https://doi.org/10.7910/DVN/V6XMJ3 GAP Year_Quantitative SRH data ^59^ The following tools are found at: https://doi.org/10.7910/DVN/AHHWNL ^60^ GAPYear_REDCap Codebook.pdf GAP Year_ACASI Boys Survey Codebook.pdf GAP Year_ACASI Girls Survey Codebook.pdf Harvard Dataverse: GAP Year_Violence REDCap and ACASI data, https://doi.org/10.7910/DVN/AHHWNL ^60^. This project contains the following extended data: GAP Year Boys ACASI Survey Questionnaire.pdf GAP Year Girls ACASI Survey Questionnaire.pdf GAP Year Boys REDCap Survey Questionnaire.pdf GAP Year Boys REDCap Survey Questionnaire.pdf Data are available under the terms of the Creative Commons Zero "No rights reserved" data waiver (CC0 1.0 Public domain dedication).

## Footnotes

^*^ Quintile 1–3 schools are non-fee-paying schools, serving the poorest communities. Quintile 1 is the group of schools in each province catering for the poorest 20% of learners and receiving the highest allocation of Government funding per learner to support these poorer schools.

^†^ Defined as a program that teaches you about sexual and reproductive health
