# Supplementary material for: Knowledge and uptake of contraceptive and other sexual reproductive health services among in-school adolescents in three South African townships: Baseline findings from the Girls Achieve Power (GAP Year) Trial
Source: Gates Open Res. 2024 Nov 11;6:67. Originally published 2022 May 25. [Version 3] doi: 10.12688/gatesopenres.13636.3 (PMC10213203; doi:10.12688/gatesopenres.13636.3)
Supplement: Peer review history

# Reviewer report: Reviewer response for version 3

**DOI:** 10.21956/gatesopenres.16764.r38445

**Published:** 2024-12-05

**Authors:** Julie Hernandez

**Affiliations:** 1 Department of Health Management and Policy, School of Public Health and Tropical Medicine, Tulane University, New Orleans, LA, USA

I reviewed and I approve the manuscript with the proposed revisions.

Is the work clearly and accurately presented and does it cite the current literature?

Yes

If applicable, is the statistical analysis and its interpretation appropriate?

Partly

Are all the source data underlying the results available to ensure full reproducibility?

Yes

Is the study design appropriate and is the work technically sound?

Partly

Are the conclusions drawn adequately supported by the results?

No

Are sufficient details of methods and analysis provided to allow replication by others?

Partly

Reviewer Expertise:

International family planning

I confirm that I have read this submission and believe that I have an appropriate level of expertise to confirm that it is of an acceptable scientific standard.

# Reviewer report: Reviewer response for version 2

**DOI:** 10.21956/gatesopenres.14980.r33165

**Published:** 2023-05-30

**Authors:** Megan A Cohen

**Affiliations:** 1 Emory University, Atlanta, Georgia, USA

This is a cross-sectional baseline study on SRH knowledge and practices of grade 8 schoolchildren in South Africa. The information was gathered as part of a larger interventional study. Knowledge and practices are important topics and relevant to readers. However, several issues remain to be strengthened.

- There is now too much information on the GAP Year intervention trial included such that it is confusing.
- I do not see any information provided on knowledge of contraceptive methods. Was there any collected? I would definitely recommend including that if so. 
- The discussion still appears to state conclusions and recommendations that are more editorializing and are not directly derived from the results. I really do not see where this was amended per the recommendation of a prior reviewer, except to add information on where the GAP Year trial aimed to address issues. 
- For example, in the paragraph starting with "school teachers and mothers were noted as the most common source of SRH information," the authors then state that an important priority is "to equip parents with the knowledge and skills to communicate effectively with their children about sex," but no information in this study actually addressed whether the students felt that their parents lacked knowledge or that they felt they could not communicate with them; that is assumed by the authors. 
- As another example, the authors mention in the discussion the need to promote availability of other contraceptive options such as the contraceptive implant and the IUD, but there is no actually information presented regarding knowledge on contraceptive methods. Therefore, it is unclear if the participants were aware of the implant as an option but chose another method instead, or if they were unaware of the methods. 
- I have some concerns regarding discrepancies in the data reported in the tables, see below. 

**Specific suggestions:**

**Methods:**

1. Too much information is reported on the GAP Year trial which confuses what this study actually entails. I suggest editing information to a general, concise overview of the trial. Particularly I would remove information on randomization as that is irrelevant to this study. 
2. The sample size justification reflects that of the actual trial. I would clearly state that. Were the outcomes of this study powered for in any way?

**Results:**

1. Table 1– how does the 12-14 year old group have 14 people with sexual debut at 15-17?
2. Table 2 – footnote a for “SRH services required” states multiple choice question not asked of males, but there are data provided for the male participants. Please explain. It would also be helpful to provide the n of the sample asked if it differs from the n=2337 listed above, but again it is not clear if this is actually a different subsample since data are presented for male participants.
3. Table 2 – What is the n of participants who were asked the question “Requested contraceptive services at last visit?”
4. There is an “*” on “Main Source of SRHR information” that does not correspond to any footnote I can see. However there are no male data presented – perhaps that was supposed to correspond to the note for footnote a “multiple choice question, not asked of males?”
5. And please clarify "multiple choice" – it appears that means “select all that apply” such that the values may surpass 100%. I believe the correct terminology should be “multiple response” or “multiple answer” instead of “multiple choice.”
6. Table 3 – please specify in the table that the question “contraceptive method ever used” is calculated based only on those who have used a contraceptive method as the denominator. For example I would consider putting the “n” in the table per age group in the same line as the heading, or list the total “n” before the footnote.
7. Table 3, footnote a – again would recommend changing to “multiple response” or “multiple answer”.
8. Table 3 “Current contraceptive method used” – it looks like this is also a multiple response question? Please clarify and specify if so.
9. Table 3 - Why is there no p-value listed for “know that condoms can prevent HIV and STIs”?
10. Table 3 - The numbers are not adding up correctly for “if no, who made the decision for you?” States n=80 but 60 answers and 100% in each group chose parents. I think it is because there are 18 missing responses, but this is confusing. In this case would change the n to be n=60.
11. The text states that “65 females were current contraceptive users” but I do not see how that reflected anywhere in Table 3 and seems to conflict with the table, I would recommend adding it to Table 3 if it was asked as a question and it is accurate. All I see is that 80 had ever used contraception, and it appears that 36 answered the question regarding current contraception used.
12. The authors state, “Of these 59% (n=36) reported current contraceptive use with two-thirds (69.4%, n=25) using the injection while a quarter (25.0%, n=9) using the condom” but the values stated for condom use directly conflict with the numbers stated in the table (58.3%, n=21).

**Discussion:**

1. I would recommend changing “experienced a lack of confidentiality” to “ *perceived* a lack of confidentiality”.
2. I would recommend revising based on recommendations in the beginning. 
3. The authors state, "final study sample size was less than originally planned however the results maintain power to draw these conclusions," but again, I do not think these outcomes and sub-analyses were powered for at all? 

Is the work clearly and accurately presented and does it cite the current literature?

Yes

If applicable, is the statistical analysis and its interpretation appropriate?

Partly

Are all the source data underlying the results available to ensure full reproducibility?

Yes

Is the study design appropriate and is the work technically sound?

Yes

Are the conclusions drawn adequately supported by the results?

Partly

Are sufficient details of methods and analysis provided to allow replication by others?

Yes

Reviewer Expertise:

sexual and reproductive health, global family planning, contraception

I confirm that I have read this submission and believe that I have an appropriate level of expertise to confirm that it is of an acceptable scientific standard, however I have significant reservations, as outlined above.

# Response

**Published:** 2024-01-25

**Authors:** Alison Kutywayo

**Affiliations:** Wits Reproductive Health and HIV Institute, South Africa

Thank you for your extensive review of our manuscript. We welcome this feedback for strengthening the paper and have addressed the comments as follows: 

**General comments: **

- Sentence added to Introduction to clarify terminology and use of the word ‘’knowledge’’.

**Methods: **

- A previous reviewer asked for more information on the GAP Year program however we have edited this section to streamline it further.
- The outcomes of the study were all powered for using a power of 0.8. However, given that there were small sample sizes in some of the variables, this is the reason why a number of variables were non-significant. An explanation of how the sample size was derived was provided on page 5. We have however indicated that it represents the actual sample of the trial. 

**Results: **

- The discrepancies in the data reported in the tables have been amended. Footnotes have been added to Table 2 to provide the reader clarity. We have removed p-values from Table 3 given the small sample size and non-significant differences.

**Discussion: **

- The Discussion has been streamlined and revised in line with the key findings.
- The Limitations have been amended, in line with the reviewers comments. 

# Reviewer report: Reviewer response for version 2

**DOI:** 10.21956/gatesopenres.14980.r33160

**Published:** 2023-05-25

**Authors:** Julie Hernandez

**Affiliations:** 1 Department of Health Management and Policy, School of Public Health and Tropical Medicine, Tulane University, New Orleans, LA, USA

Thank you for the opportunity to review this manuscript, which provides additional contributions to the literature on early adolescents’ SRH issues in South Africa. The study design and papers are overall clear and well developed. However, the reviewer thinks that some revisions are necessary to ensure the findings are rigorously presented and the recommendations align with the study results.

 Key cross-sectional issues include:

- The need to clarify between contraceptive use for pregnancy prevention vs. condom use for STI (including HIV) prevention. The authors mention “SRH and HIV” but the latter is included in the former. The results would benefit from clarifying the specific issues faced by young adolescents with regards to both contraceptive use and STI prevention.
- “Knowledge” is not well defined in the Methods section and as a result it is often unclear whether actual knowledge (e.g. including of how to use, possible side effects, etc.) or simply method awareness was recorded. In addition, the authors should clarify whether they recorded method awareness through direct “yes / no” questions (“Have you ever heard of method (x)?”) or by asking respondents to list the methods they knew?
- Method knowledge is not reported in any of the tables despite figuring prominently in the introduction, the key outcomes and the title for the study.
- Since participation was voluntary, what was the refusal rate / participation rate for each school, and does this introduce a potential for bias?
- Since the denominator for women who ever had sex dropped considerably (n = 157), could the authors comment on the power to run the association analysis presented in Table 3? Would it be possible to raise the significance threshold to counter the small denominator issue? Results are presented in details (p.9) and yet those specific samples are very small.
- Finally, as mentioned by another reviewer, the Discussion section still stretches the results considerably. Details are provided under suggestions for this specific section.

 

 I also suggest the following edits to each section:

**Introduction:**

- Section 2: Adjust “SRH and HIV”. SRH includes HIV / AIDS / STI and contraceptive access and use.
- Section 3: “the prevalence of mostly unintended pregnancies” Suggest reformulating, unclear what “mostly” means here, nor what data supports it.
- Section 5: First sentence is circumvoluted, I suggest reformulating.

 

**Table 1:**

****

 Significant difference for currently dating / ever had sex by girls might be indicative of reporting bias and needs to be discussed in Limitations. Literature consistently shows that adolescent girls underreport sexual activity.

**Table 2**

 Significant difference in “ever participated in an SRH program” for girls indicates that girls are much more frequently targeted by this kind of program and should be discussed in relevant section (esp. as authors advocate for gender-based interventions).

 It might also explain why girls are significantly more likely than boys to access youth clinics (because SRH programs may have made them aware of their availability).

 Table 2 presents knowledge of contraceptive services but not of contraceptive methods. Manuscript title, background and methods section should be adjusted to reflect this.

**Table 3:**

 Is “Thigh sex” a traditional method? In that case it should be lumped with “Withdrawal” and other non-modern contraceptive methods.

**Discussion:**

 I re-iterate the comment of a previous reviewer who found the discussion and conclusion too generic and not sufficiently aligned with the specific findings of the study.

 The first section does not reflect the specific findings presented in the previous section. For example, looking at the different age groups (since this is an important point highlighted by the authors in the recommendations) Table 1 and 2 suggest that older adolescents are more likely to have been exposed to SRH programs (which makes sense considering they have been alive / in the target demographic for a longer period of time), yet they are less comfortable asking SRH questions from service providers. Regardless of these differences in SRH exposure though, Table 3 indicates that there are no significant differences in use patterns between age groups.

 The authors do not discuss supply-side issues and barriers that may challenge their proposed recommendations. What factors could limit the feasibility of implementing those recommendations considering the resource-limited health provision environment of South African townships?

 Section 3 and 4 of the Discussion presents the GAP Year as a solution to the issues highlighted in the manuscript which might read as confirmation bias, particularly as the study uses only pre-intervention data. We suggest removing or reformulating to present how GAP Year specifically addresses these issues.

 The last three paragraphs of this section present conclusions that are not based on the study findings. For example, data about participants thinking that hormonal contraception can prevent STI / HIV is not mentioned in any of the previous sections.

 It might be more appropriate to talk about “respondents” rather than “learners” presuming that not all students participated in the survey? (Participation bias).

 The last section could also be construed as confirmation bias in advocating for ecological models such as the one used by GAP Year.

**Strengths and limitations:**

 The sample of 26 schools was not selected in any representative way (or the randomization technique was not adequately presented in the Methods), therefore the results can **NOT** be generalized to other South African settings. (Large samples are not sufficient to generate external validity).

Is the work clearly and accurately presented and does it cite the current literature?

Yes

If applicable, is the statistical analysis and its interpretation appropriate?

Partly

Are all the source data underlying the results available to ensure full reproducibility?

Yes

Is the study design appropriate and is the work technically sound?

Partly

Are the conclusions drawn adequately supported by the results?

No

Are sufficient details of methods and analysis provided to allow replication by others?

Partly

Reviewer Expertise:

International family planning

I confirm that I have read this submission and believe that I have an appropriate level of expertise to confirm that it is of an acceptable scientific standard, however I have significant reservations, as outlined above.

# Response

**Published:** 2024-01-25

**Authors:** Alison Kutywayo

**Affiliations:** Wits Reproductive Health and HIV Institute, South Africa

Thank you to the reviewer for this thoughtful and thorough review of our manuscript. There were a number of comments which have been addressed in the following ways: 

 - The use of the term ''SRH and HIV'': In South Africa, and sub-Saharan Africa, the term SRH and HIV is commonly used as a package of distinct but interrelated interventions be it prevention, treatment, health promotion, advocacy etc. While there is an intersection, the two have separate policies and programmes in South Africa, and therefore we tend to refer to SRH and HIV. The introduction provides detail of the scope in this context.

 - Regarding the definition of knowledge: We wanted to probe their knowledge and uptake about SRH services broadly; and their utilisation and experience thereof. It was beyond the scope to probe detailed knowledge about each method.  Sentence added to Introduction to clarify terminology and use of the word ‘’knowledge’’.

 - Based on reviewer feedback, the following limitations have been added: limited generalizability due to small school sample, refusal rate not collected leading to possible participation bias and some significant differences presented in the results may be attributed to under-reporting and AGYW targeting. 

 - Power to run the study: Prior to commencing with our analysis, we set the statistical power to 0.8 (can also be specified as 80%) with an effect size of 5% and an alpha of 0.05 and this is usually considered to be the ideal power of a study. This was done with the purpose of lowering the risk of making a Type II error and thus given this instance, it was important to rather lean towards a Type I error which was as low as 0.05 or 0.01. We do however acknowledge that the sample size of young women who have ever had sex (n=157) was not sufficient enough to conduct inferential statistics with which affects the ability to comment on any significant differences between the two age groups. The n presented is lower (n=80) as it includes only information on the contraceptive users. We have therefore removed the P values which were non-significant and mentioned the descriptive presentation when the table is introduced.

 - Minor edits to the text have been amended, as per the reviewers suggestions. 

 - Table 3: uptake of contraception methods is presented in Table 3.

 - Discussion: the Discussion has been edited and streamlined extensively aligning to the key findings, as suggested by the reviewer.

 - Discussion and Conclusion has been reviewed to ensure recommendations acknowledge the resource constrained context within which this research was conducted. 

 - References made to the GAP Year intervention have been removed in the Discussion. 

 -  Throughout the manuscript we now talk about ''participants'', not learners. 

 Please see the updated version of the manuscript for your review.

# Reviewer report: Reviewer response for version 2

**DOI:** 10.21956/gatesopenres.14980.r32262

**Published:** 2022-07-19

**Authors:** Catherine MacPhail

**Affiliations:** 1 School of Health and Society, University of Wollongong, Wollongong, NSW, Australia

I have no further comments to make.

Is the work clearly and accurately presented and does it cite the current literature?

Yes

If applicable, is the statistical analysis and its interpretation appropriate?

I cannot comment. A qualified statistician is required.

Are all the source data underlying the results available to ensure full reproducibility?

Yes

Is the study design appropriate and is the work technically sound?

Yes

Are the conclusions drawn adequately supported by the results?

Yes

Are sufficient details of methods and analysis provided to allow replication by others?

Yes

Reviewer Expertise:

adolescent sexual and reproductive health, particularly HIV prevention

I confirm that I have read this submission and believe that I have an appropriate level of expertise to confirm that it is of an acceptable scientific standard.

# Reviewer report: Reviewer response for version 1

**DOI:** 10.21956/gatesopenres.14915.r32119

**Published:** 2022-06-20

**Authors:** Catherine MacPhail

**Affiliations:** 1 School of Health and Society, University of Wollongong, Wollongong, NSW, Australia

This paper reports on the baseline findings from an intervention programme to increase contraceptive knowledge and use among young South African adolescents in three township communities.

 Although the GAP Year intervention is included in the title of the paper, there is no real mention of the intervention in the paper. Would it be possible to include some details of the proposed intervention (likely completed already given the dates of this baseline data collection)? It seems that including some ideas of how the GAP Year intervention might address some of the issues outlined in the discussion would enhance the paper by reporting on how the findings might be addressed in the intervention.

**Abstract:**

- Remove the apostrophe from Grade 8’s

**Introduction:**

- Suggest adding a comma to “there are still multiple challenges in accessible SRH service provision for adolescents, resulting in poor health outcomes.”
- “limited information on contraception knowledge” seems to be convoluted language and might benefit from some additional editing
- Change “Knowledge about contraception and contraceptive options is low among South African youth,. . .”

**Methods:**

- The recruitment of Grade 8 participants was undertaken across two school years. The authors might wish to comment on whether this was an intentional plan (and to what end) or to comment on any implications on this method in the limitations section.
- It is interesting that sensitive questions about sexuality, gender, and norms were included in the interviewer-administered questionnaire component and not in the ACASI component. I assume that this was because these were general questions and not specifically about the participant’s own identity?
- Please comment on the use of a Xhosa translation for the W Cape schools but no equivalent translation for the Gauteng schools. What was the reasoning behind this decision? This should potentially be further discussed in the limitations section if you believe that this might have impacted on the accuracy of information or completion. It would be useful here to see completion by province – given that quite a large number of students did not complete both parts of the survey, might language problems have contributed in the Gauteng cohort?

**Results:**

- Table 2 results note that there is a significant difference in knowledge of right to accessing health care by gender (p<0.001), yet the reporting in the text says “Over two thirds of learners (66.8%, n=1557) perceived that they have a right to access health care without being discriminated or stigmatised by health workers: this was similar among males and females.” This doesn’t seem consistent.

Is the work clearly and accurately presented and does it cite the current literature?

Yes

If applicable, is the statistical analysis and its interpretation appropriate?

I cannot comment. A qualified statistician is required.

Are all the source data underlying the results available to ensure full reproducibility?

Yes

Is the study design appropriate and is the work technically sound?

Yes

Are the conclusions drawn adequately supported by the results?

Yes

Are sufficient details of methods and analysis provided to allow replication by others?

Yes

Reviewer Expertise:

adolescent sexual and reproductive health, particularly HIV prevention

I confirm that I have read this submission and believe that I have an appropriate level of expertise to confirm that it is of an acceptable scientific standard.

# Response

**Published:** 2022-07-01

**Authors:** Alison Kutywayo

**Affiliations:** Wits Reproductive Health and HIV Institute, South Africa

Thank you for your comments on our manuscript. 

 We have noted your comments and have addressed them as follows: 

 Although the GAP Year intervention is included in the title of the paper, there is no real mention of the intervention in the paper. Would it be possible to include some details of the proposed intervention (likely completed already given the dates of this baseline data collection)? It seems that including some ideas of how the GAP Year intervention might address some of the issues outlined in the discussion would enhance the paper by reporting on how the findings might be addressed in the intervention.

- **Response**: Added a sentence in the Methods section to elaborate on the GAP Year intervention and then also mentioned the intervention in the Discussion, in line with this comment.  

Abstract: Remove the apostrophe from Grade 8’s

- **Response**: Apostrophe removed.

Introduction: Suggest adding a comma to “there are still multiple challenges in accessible SRH service provision for adolescents, resulting in poor health outcomes.”

- **Response**: Punctuation added.

Intro: “limited information on contraception knowledge” seems to be convoluted language and might benefit from some additional editing

- **Response**: Punctuation added.

Intro: Change “Knowledge about contraception and contraceptive options is low among South African youth…

- **Response**: Sentence amended.

Methods: The recruitment of Grade 8 participants was undertaken across two school years. The authors might wish to comment on whether this was an intentional plan (and to what end) or to comment on any implications on this method in the limitations section

- **Response**: This section in the Methods has been amended and clarity provided.

Methods: It is interesting that sensitive questions about sexuality, gender, and norms were included in the interviewer-administered questionnaire component and not in the ACASI component. I assume that this was because these were general questions and not specifically about the participant’s own identity?

- **Response**: This section of the Methods has been restructured to provide greater clarity on this query.   

Methods: Please comment on the use of a Xhosa translation for the W Cape schools but no equivalent translation for the Gauteng schools. What was the reasoning behind this decision? This should potentially be further discussed in the limitations section if you believe that this might have impacted on the accuracy of information or completion. It would be useful here to see completion by province – given that quite a large number of students did not complete both parts of the survey, might language problems have contributed in the Gauteng cohort?

- **Response**: This section in the Methods has been amended to provide clarity on the translation.

Results: Table 2 results note that there is a significant difference in knowledge of right to accessing health care by gender (p<0.001), yet the reporting in the text says “Over two thirds of learners (66.8%, n=1557) perceived that they have a right to access health care without being discriminated or stigmatised by health workers: this was similar among males and females.” This doesn’t seem consistent

- **Response**: Although the difference between females (65.2% vs male 69.5%) table 2 appears to be a small percentage and similar, the large sample size indicates that statistically there is a real difference between the males and females in this variable. We have changed the text to make this more clear.

# Reviewer report: Reviewer response for version 1

**DOI:** 10.21956/gatesopenres.14915.r32124

**Published:** 2022-06-08

**Authors:** Belete Yimer

**Affiliations:** 1 Department of Human Nutrition, Debre Markos University, Debre Markos, Ethiopia

- The document makes important, relevant points, is timely and contains applicable references. I agree with much of the analysis, but think it needs strengthening. 
- The title should be modified as: "Contraceptive knowledge and uptake among school adolescents in three South African townships...", in order to better convey the key features of the article.
- The authors used Chi-squared statistic tests for proportion comparison. To identify predictors of interest of knowledge, uptake and perceptions around SRH controlling for the confounding effects, multiple regression could be used.
- The recommendations need to be based on the findings of the study in question and not general observations and dissemination of knowledge. I suggest the authors to narrow down their recommendations and focus on what they found alone.
- I feel the study holds merit and may be of scientific interest. However I feel the above observations need to be addressed.

Is the work clearly and accurately presented and does it cite the current literature?

Yes

If applicable, is the statistical analysis and its interpretation appropriate?

No

Are all the source data underlying the results available to ensure full reproducibility?

Yes

Is the study design appropriate and is the work technically sound?

Yes

Are the conclusions drawn adequately supported by the results?

Partly

Are sufficient details of methods and analysis provided to allow replication by others?

Yes

Reviewer Expertise:

Maternal and child health and nutrition

I confirm that I have read this submission and believe that I have an appropriate level of expertise to confirm that it is of an acceptable scientific standard, however I have significant reservations, as outlined above.

# Response

**Published:** 2022-07-01

**Authors:** Alison Kutywayo

**Affiliations:** Wits Reproductive Health and HIV Institute, South Africa

The title should be modified as: "Contraceptive knowledge and uptake among school adolescents in three South African townships...", in order to better convey the key features of the article.

- **Response**: The title has been amended as suggested.

The authors used Chi-squared statistic tests for proportion comparison. To identify predictors of interest of knowledge, uptake and perceptions around SRH controlling for the confounding effects, multiple regression could be usedThe authors used Chi-squared statistic tests for proportion comparison. To identify predictors of interest of knowledge, uptake and perceptions around SRH controlling for the confounding effects, multiple regression could be used

- **Response: **The data presented is from the baseline- prior to any intervention. We were not expecting high levels of service uptake in this group of grade 8 learners. Grade 8 learners are usually aged 12-14 years but there were a significant proportion who were older (15-18) and these young people would have had to repeat school years due to exam failure or other social/financial/health reasons for school exclusion. In terms of their maturity, sexual experience and social circumstances they are a very different group and so we tried to focus on these as two separate and distinct age groups and not compare them too closely at baseline due to their different circumstances. In the 12 -14 age group although table 2 shows 10% of females had ever had sex - a much lower proportion had experienced sexual debut in the 12-14 age compared to the 15-18 year olds as expected. SRH service uptake was also extremely low in both age groups and in males and females which we expected at this age as few were sexually experienced. Only 11 girls aged 12-14 of 1504 had accessed HIV testing services for instance. Because of this low uptake, we felt that a descriptive analysis would be easier to present as numbers were too low for any meaningful multiple regression or other more complex analysis.

The recommendations need to be based on the findings of the study in question and not general observations and dissemination of knowledge. I suggest the authors to narrow down their recommendations and focus on what they found alone.

- **Response**: In addressing this comment, we have removed the general observations in the Discussion and Conclusion to ensure it is inline with our findings.
